# Molecular probes for the human adenosine receptors

**DOI:** 10.1007/s11302-020-09753-8

**Published:** 2020-12-12

**Authors:** Xue Yang, Laura H. Heitman, Adriaan P. IJzerman, Daan van der Es

**Affiliations:** Division of Drug Discovery and Safety, Leiden Academic Centre for Drug Research, Leiden University, Einsteinweg 55, 2333 CC Leiden, The Netherlands

**Keywords:** Probes, Adenosine receptors, GPCR, Chemical biology, Radioligands, PET ligands, Fluorescent ligands, Covalent ligands

## Abstract

Adenosine receptors, G protein–coupled receptors (GPCRs) that are activated by the endogenous ligand adenosine, have been considered potential therapeutic targets in several disorders. To date however, only very few adenosine receptor modulators have made it to the market. Increased understanding of these receptors is required to improve the success rate of adenosine receptor drug discovery. To improve our understanding of receptor structure and function, over the past decades, a diverse array of molecular probes has been developed and applied. These probes, including radioactive or fluorescent moieties, have proven invaluable in GPCR research in general. Specifically for adenosine receptors, the development and application of covalent or reversible probes, whether radiolabeled or fluorescent, have been instrumental in the discovery of new chemical entities, the characterization and interrogation of adenosine receptor subtypes, and the study of adenosine receptor behavior in physiological and pathophysiological conditions. This review summarizes these applications, and also serves as an invitation to walk another mile to further improve probe characteristics and develop additional tags that allow the investigation of adenosine receptors and other GPCRs in even finer detail.

## Introduction

Adenosine receptors (ARs) belong to the class A family of G protein–coupled receptors (GPCRs) and are activated by their endogenous ligand adenosine. These receptors have been considered potential therapeutic targets in several disorders, including Parkinson’s disease, schizophrenia, analgesia, ischemia, and cancer [1]. To date, four subtypes of adenosine receptors have been identified, namely A_1_, A_2A_, A_2B_, and A_3_. Activation of A_1_ and A_3_ receptors leads to inhibition of adenylate cyclase through their interaction with a Gα_i_ protein, whereas A_2A_ and A_2B_ receptors stimulate the enzyme through a Gα_S_-linked pathway. Until now, the 3D structures of the A_1_ and A_2A_ subtypes have been elucidated [2, 3]; structural studies on the A_2B_ and A_3_ subtypes have yet to be successful. Crystallization of GPCRs, often a prerequisite for structural biology, still proves to be a challenging task due to their low expression in native tissue, and their inherent flexibility and instability once extracted from the membrane, which is needed for further structural studies. Over the past decades, a diverse array of molecular probes, bifunctional ligands that can be used to interrogate receptor structure and function, has proven invaluable in GPCR research. From a chemical perspective, a molecular probe can be defined as a small molecule that binds the receptor of interest and enables further studies by virtue of a connected tag or functional group that exhibits specific properties. These conjugated tags or functional groups include radioactive or fluorescent moieties to enable studies on ligand–receptor binding as well as the quantification and visualization of receptors. Moreover, tags containing a reactive warhead capable of irreversibly binding to the receptor have been shown to facilitate structure elucidation. When made bifunctional, i.e., combined with a click handle, these tags can be used as *affinity*-based probes (AfBPs), which are emerging as valuable tools for chemical biology or proteomics studies to gain further insight into receptor localization and target engagement [4–6]. This strategy was inspired by earlier *activity*-based protein profiling-click chemistry (ABPP-CC), which helped in visualizing and quantifying the activities of drug targets (mainly enzymes) in native biological systems [7, 8]. In this review, various chemical probes for human adenosine receptors, comprising radioligands, fluorescent ligands, and covalent ligands, will be summarized.

## Radioligands for in vitro receptor characterization

Some adenosine receptor agonists and antagonists have been developed in a radiolabeled (“hot”) form, so-called radioligands. Often, these are high-affinity molecules containing radioactive isotopes such as [^3^H]-, [^125^I]-, and [^35^S]-, which emit radiation that can be detected and quantified. The majority of radioligands used for in vitro assays are labeled with either [^125^I] or [^3^H]. While [^125^I]-labeled ligands show a higher specific activity (∼ 2000 Ci/mmol) and shorter half-life (*t*_1/2_ = 60 days) compared to tritium-labeled ligands (specific activity ~ 25–120 Ci/mmol and *t*_1/2_ = 12.5 years), [^3^H]-labeled compounds are more biologically indistinguishable from the unlabeled parent ligand. These radiolabeled ligands are predominantly used in (i) saturation experiments to measure the radioligand’s equilibrium dissociation constant, *K*_*D*_, and receptor expression/density (*B*_max_); in (ii) competition displacement experiments to determine the affinity (equilibrium inhibitory constant *K*_*i*_) of non-labeled (“cold”) compounds; and in (iii) binding kinetics assays to determine a ligand’s association (*k*_on_) and dissociation *(k*_off_*)* rate constants [9, 10]. Conventional radioligand binding assays require a filtration step to separate bound from unbound radiolabeled ligands and capture the radioligand–receptor complex. A more recently developed bead-based assay, the scintillation proximity assay (SPA), has emerged as a rapid and sensitive assay to perform high-throughput screens in a homogeneous system. Due to the diverse applicability of these techniques in receptor research, a diverse set of radioligands for the different AR subtypes has been developed. All radioligands that are currently commonly used are summarized in Table 1.

**Table 1:** Commonly used AR radioligands for in vitro studies

Radioligands	*K*_*D*_^a^ (nM)	Functionality	Refs	Commercially available
A_1_				
[^3^H]CCPA	0.61	Agonist	[11]	N
[^3^H]LUF5834	2.0	Agonist	[12, 13]	N
[^3^H]DPCPX	3.9	Antagonist	[11]	Y
A_2A_				
[^3^H]NECA	20	Agonist	[11]	Y
[^3^H]CGS21680	22	Agonist	[14]	Y
[^3^H]XAC	9.4	Antagonist	[15]	N
[^3^H]MSX-2	8.0	Antagonist	[16]	Y
[^3^H]ZM241385	0.60	Antagonist	[17]	Y
[^3^H]SCH58261	2.3	Antagonist	[18]	Y
A_2B_				
[^3^H]NECA	441	Agonist	[19]	Y
[^3^H]DPCPX	40	Antagonist	[20]	Y
[^125^I]I-ABOPX	37	Antagonist	[21]	N
[^3^H]MRS1754	1.1	Antagonist	[22]	Y
[^3^H]MRE-2029-F20	2.8	Antagonist	[23]	Y
[^3^H]OSIP339391	0.17	Antagonist	[24]	N
[^3^H]PSB-603	0.40	Antagonist	[25]	N
A_3_				
[^3^H]NECA	6.2	Agonist	[11]	Y
^125^I-APNEA	15 (r)	Agonist	[26]	N
[^125^I]I-AB-MECA	1.9	Agonist	[27]	Y
[^3^H]HEMADO	1.1	Agonist	[28]	Y
[^125^I]MRS1898	0.17 (r)	Agonist	[29]	N
[^125^I]MRS5127	5.7	Partial agonist	[30]	N
[^3^H]MRE-3008-F20	0.80	Antagonist	[31]	N
[^3^H]PSB-11	4.9	Antagonist	[32]	N

^a^The data are *K*_*D*_ values for radiolabeled compounds (nM) for the indicated human adenosine receptors unless a different species is indicated (r = rat)

### Radioligands for the adenosine A_1_ receptor

**Fig. 1:**
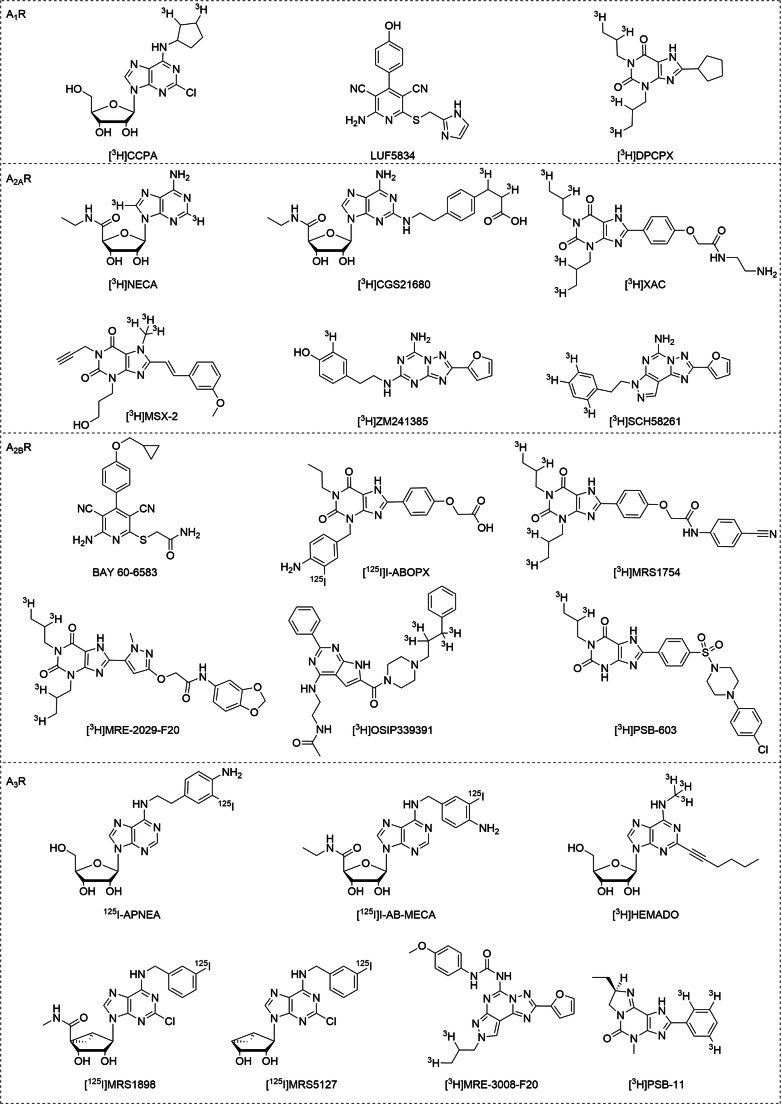
Chemical structures of commonly used AR radioligands for in vitro studies. Unlabeled version was drawn for radioligands with unknown radioisotope position (i.e., [^3^H]LUF5834 and [^3^H]BAY 60-6583)

Starting with agonist radioligands for A_1_R, initially only tritiated adenosine-based derivatives were developed. Among them, [^3^H]CCPA (Fig. 1; Table 1) showed the highest affinity with a *K*_*D*_ value of 0.61 nM for human A_1_R (hA_1_R) [33]. [^3^H]LUF5834 is a non-nucleoside partial agonist radioligand (Fig. 1; Table 1) with nanomolar affinity (*K*_*D*_ = 2.03 ± 0.52 nM) for the hA_1_R [12]. Its partial agonistic nature allows this radioligand to bind to both G protein–coupled and –uncoupled receptors. This radioligand proved a versatile tool to estimate the efficacy and the mechanism of action of both agonists and inverse agonists at the hA_1_R.

The reference antagonist radioligand for A_1_R is the xanthine-derived antagonists/inverse agonist [^3^H]DPCPX (Fig. 1; Table 1) [11]. Although this xanthine derivative displays lower affinity at the human (*K*_*D*_ = 3.86 nM) [11] than the rat receptor (*K*_*D*_ = 0.18 nM) [34], it is still a very useful tool for the characterization of A_1_R and to consequently discriminate from other subtypes. It has been applied in SPA technology, constituting an alternative platform for real-time measurements of receptor–ligand interactions on hA_1_R [35]. Antagonist radioligands, contrary to agonists, tend to label all receptors present in a cell membrane preparation independent of their coupling to a G protein and are therefore used more frequently in AR research, and GPCR research in general.

### Radioligands for the adenosine A_2A_ receptor

The reference radioligands for binding assays at A_2A_R include the adenosine-based agonists [^3^H]NECA (Fig. 1; Table 1) [11] and [^3^H]CGS21680 (Fig. 1; Table 1) [36]. While [^3^H]NECA bound to hA_2A_R with a *K*_*D*_ value of 20 nM, this non-selective radioligand also exhibited remarkably high affinity for hA_3_R with a *K*_*D*_ value of 6 nM, threefold higher than at the A_2A_ receptor [11]. Later, the more selective radioligand [^3^H]CGS21680 showed a moderate affinity for human A_2A_R with a *K*_*D*_ value of 22 nM and has been used in autoradiographic studies, revealing the distribution of the A_2A_R in the basal ganglia of the human brain and an increased hA_2A_R level in the striatum of schizophrenic patients [14, 37, 38]. However, besides its agonistic binding to high- and low-affinity states of the receptor, application of this agonist radioligand is further limited due a limited selectivity over the A_3_R (*K*_*i*_ = 67 nM). This resulted in complex binding characteristics related to cortical, non-A_2A_ binding sites [39].

To avoid the issues occurring with agonistic radioligands, two xanthine-based antagonist radioligands [^3^H]XAC (Fig. 1; Table 1) [15] and [^3^H]MSX-2 (Fig. 1; Table 1) [16] were developed to investigate the A_2A_R. Although the unlabeled compound XAC showed poor selectivity for hA_2A_R over hA_1_R (30-fold) and hA_3_R (90-fold) [11], [^3^H]XAC was used to label the hA_2A_R-binding pocket with a *K*_*D*_ value of 9.4 nM [15]. [^3^H]MSX-2 is a styrylxanthine-based antagonist which bound selectively to rA_2A_R (*K*_*D*_ = 8.0 nM) [16]. Furthermore, in vitro autoradiography with [^3^H]MSX-2 showed the greatest binding in the striatum, which is in line with the expected density of A_2A_R in the mouse, rat, and pig brains [40]. A preliminary ex vivo study confirmed that [^3^H]MSX-2 penetrated the blood–brain barrier, which is promising for in vivo use [40]. Applications of these styrylxanthine derivatives are limited however, due to the tendency to undergo photo-induced isomerization [41]. Meanwhile, two non-xanthine antagonist radioligands were developed as well. [^3^H]ZM241385 (Fig. 1; Table 1) showed a high affinity and low non-specific binding to hA_2A_R [17, 42]. However, this radioligand also binds to A_2B_R with nanomolar affinity (*K*_*D*_ = 33.6 nM) [43]. [^3^H]SCH58261 (Fig. 1; Table 1) showed a better selectivity at the hA_2A_R (hA_2B_/hA_2A_ = 8352) than [^3^H]ZM241385 and was used in autoradiographic studies to investigate the receptor distribution in the human brain [18, 37]. Similarly, [^3^H]SCH58261 was applied in ex vivo binding studies to study A_2A_R receptor occupancy of various ligands in mouse brain [44]. Additionally, this radioligand was applied in high-throughput ligand screening using a SPA setup and showed comparable sensitivity to the conventional filtration assay [45].

### Radioligands for the adenosine A_2B_ receptor

So far only one selective agonist radioligand has been described for the A_2B_R, which is tritium-labeled BAY 60-6583 (Fig. 1; Table 1) [19]. Unfortunately, the specific binding of [^3^H]BAY 60-6583 was too low compared to its high non-specific binding to establish a robust radioligand binding assay. Until now, the non-selective agonist radioligand [^3^H]NECA, despite its low affinity, remains the only molecular tool available to specifically study the active A_2B_R conformation [19, 46].

The A_1_R radioligand [^3^H]DPCPX (Fig. 1; Table 1) was also reported to bind hA_2B_R (*K*_*D*_ = 40 nM) and has been used to determine the affinity of competing ligands [20, 47]. Another non-selective radioligand is [^125^I]I-ABOPX (Fig. 1, Table 1) [21], which bound to A_2B_R with moderate affinity (*K*_*D*_ = 37 nM) and showed a high specific binding to a hA_2B_R overexpressing cell line. The first A_2B_R-selective antagonist radioligand reported was [^3^H]MRS1754 (Fig. 1; Table 1), which bound to hA_2B_R with a *K*_*D*_ value of 1.1 nM [22]. Later, another xanthine analog radioligand [^3^H]MRE-2029-F20 was reported with comparable affinity and selectivity [23, 48]. The pyrrolopyrimidine-derivative OSIP339391 (Fig. 1; Table 1) was also labeled with tritium, representing a novel selective and high-affinity radioligand for the hA_2B_R [24]. However, all these radioligands showed poor selectivity (less than 100-fold) towards the hA_1_R. More recently, Müller et al. investigated the structure–activity relationships of 1-alkyl-8-(piperazine-1-sulfonyl)phenylxanthine derivatives, yielding a new and potent A_2B_-selective antagonist, PSB-603 [25]. Tritium-labeled PSB-603 (Fig. 1; Table 1) was subsequently developed and employed as the first high-affinity (*K*_*D*_ = 0.40 nM) A_2B_R-specific radioligand for receptor pharmacological studies. However, the current xanthine-based radioactive tracers are highly lipophilic compounds that exhibit unfavorable non-specific to specific binding ratios; this feature confines their application to receptor studies in isolated membranes.

### Radioligands for the adenosine A_3_ receptor

Initially, studies on the human A_3_R (hA_3_R) were performed using the non-selective agonist radioligand [^3^H]NECA (Fig. 1, Table 1) [11]. For binding studies on the rat A_3_R (rA_3_R) however, ^125^I-APNEA (Fig. 1, Table 1) was the preferred radioligand [49]. Although ^125^I-APNEA showed reasonable affinity for the rA_3_R (*K*_*D*_ = 15 nM), it was shown to be even more potent for the rA_1_R (*K*_*D*_ = 1.3 nM) [26, 49]. Another agonist radioligand, [^125^I]I-AB-MECA (Fig. 1; Table 1), showed better affinities for both rA_3_R (*K*_*D*_ = 1.5 nM) and hA_3_R (*K*_*D*_ = 1.9 nM) [26, 27], but still bound to rA_1_R in the nanomolar range (*K*_*D*_ = 3.4 nM) [26]. To tackle the selectivity challenge, Klotz et al. developed the tritiated agonist radioligand [^3^H]HEMADO (Fig. 1, Table 1) [28], which showed high-affinity (*K*_*D*_ = 1.1 nM) and low non-specific binding (1–2% at *K*_*D*_ value) to hA_3_R. Even though no binding on the rat rA_3_R was observed, the enhanced selectivity versus other AR subtypes (> 300 fold) made [^3^H]HEMADO a useful tool for A_3_R binding assays. Subsequent efforts in finding a selective ligand for the rA_3_R resulted in [^125^I]MRS1898 (Fig. 1; Table 1), which selectively binds to rA_3_R with an improved *K*_*D*_ value of 0.17 nM [29]. Still, there are some liabilities caused by the high non-specific binding. The truncation of the 5′-position of the ribose moiety generated the latest A_3_R agonist radioligand [^125^I]MRS5127 (Fig. 1; Table 1) with a *K*_*D*_ value of 5.7 nM [30]. Its major advantage is the low degree of non-specific binding (27 ± 2% at a concentration of 5 nM) and its improved selectivity versus the other AR subtypes. These benefits, together with the uniformity of its agonistic nature across species, may render [^125^I]MRS5127 the preferred chemical tool for characterizing the A_3_R in its active state over other radioligands reported previously. Commercially available [^125^I]I-AB-MECA has emerged as a reference radioligand though.

Until now, only two antagonist radioligands, [^3^H]MRE-3008-F20 (Fig. 1; Table 1) [31, 50] and [^3^H]PSB-11 (Fig. 1; Table 1) [32], have been reported for the A_3_R. While both derivatives selectively bind the hA_3_R at (sub)nanomolar concentrations, [^3^H]PSB-11 shows a much lower degree of non-specific binding (2.5 ± 0.1% at *K*_*D*_ value) than [^3^H]MRE-3008-F20 (ca. 25% at *K*_*D*_ value). The downside of these structurally diverse heterocyclic antagonists is their low affinity for the A_3_R in non-human, particularly rodent tissue.

## Radioligands for in vivo studies—PET/SPECT tracers

While β-emitting ligands serve their purpose in in vitro or ex vivo experiments, they are not suitable for in vivo application. To that end, positron emission tomography (PET) and single-photon emission computed tomography (SPECT) scanning have emerged and are noninvasive quantitative techniques to measure the receptor distribution and function in vivo. Over the years, an ever-expanding library of [^11^C]-, [^18^F]-, and [^123^I]-labeled radiotracers has been developed that enables the determination of receptor binding potentials (BPs) in physiological and pathophysiological studies. Although the decay of these isotopes is much faster than is the case for [^3^H]- or [^125^I]-labeled ligands, the relatively safe *γ*- and photon-emissions make these tracers suitable for physiological applications. SPECT radioisotopes, such as *γ*-emitting [^123^I] (*t*_1/2_ = 13.2 h), typically have a much longer half-life than PET tracers labeled with [^11^C] (*t*_1/2_ = 20.3 min) or [^18^F] (*t*_1/2_ = 110 min), which allow for longer radiosynthetic protocols and enable SPECT imaging to be conducted for longer time periods. Nonetheless, PET studies of adenosine receptors have been more widely performed due to the higher resolution and sensitivity that can generally be achieved compared to SPECT. In the development of radiotracers for ARs, particularly in the brain and central nervous system, it is desirable to not only optimize for affinity and low non-specific binding capacity, but also for blood–brain barrier permeability. A major challenge is that the short radioligand half-life requires on-site synthesis and rapid purification and validation of the probes. PET and SPECT imaging times, which are also related to radioligand *t*_1/2_, are usually insufficient to allow radioligand–receptor binding to reach an equilibrium; therefore, appropriate kinetic models should be used to correct for this shortcoming. PET imaging of ARs in vivo and the applications thereof in drug discovery have been comprehensively reviewed [51–53]. Here, we will focus on the recent applications of clinical PET imaging studies on ARs.

### PET tracers for the adenosine A_1_ receptor

**Fig. 2:**
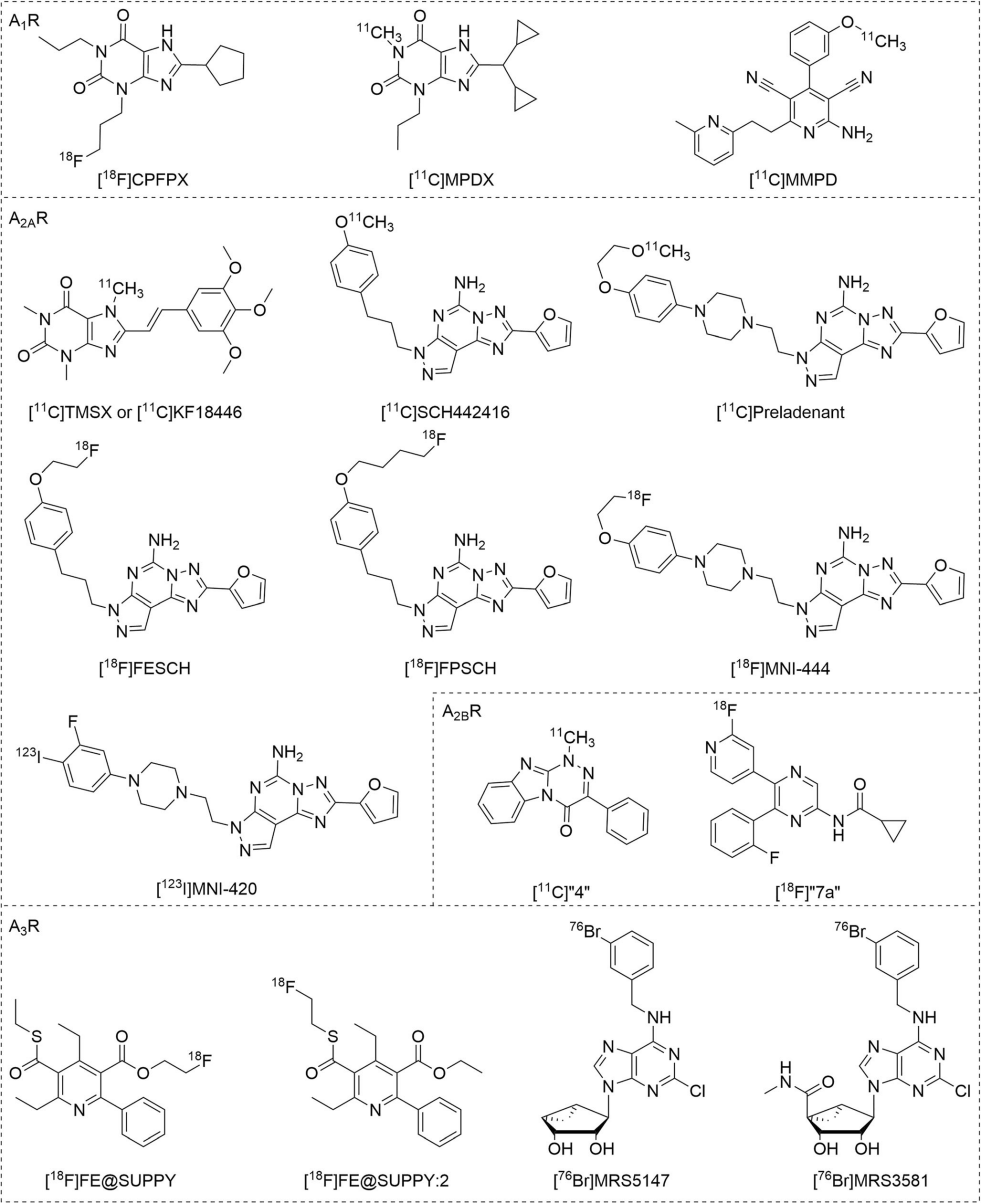
Chemical structures of AR radioligand tracers for in vivo studies

Two xanthine derivatives, [^18^F]CPFPX (Fig. 2, Table 2) and [^11^C]MPDX (Fig. 2, Table 2), have been extensively employed for the characterization of A_1_R in human brain, and their results are summarized in several reviews [51, 65]. While [^18^F]CPFPX has a higher affinity for A_1_R than [^11^C]MPDX, the latter has been shown to be much more stable against peripheral metabolism. Using these PET tracers, the cerebral distribution of the A_1_R has been successfully visualized and quantified in human brain [66, 67]. From these studies, a correlation between A_1_R distribution and aging as well as sleep deprivation was established [68, 69]. Additional studies on receptor occupancy using PET tracers, for example [^18^F]CPFPX in a bolus-plus-constant-infusion PET assay, showed that repeated intake of caffeinated beverages resulted in a 50% occupancy of the cerebral A_1_Rs during the day [70]. This effect might cause adaptive changes and lead to chronic alterations of receptor expression and availability. Furthermore, these PET tracers have been valuable tools for clinical studies on neurodegenerative diseases, revealing the functional mechanisms and pharmacokinetic profiles of new potential drug treatment strategies. In early Parkinson’s disease, increased binding of [^11^C]MPDX was found in the temporal lobe, suggesting a compensatory mechanism of A_1_R expression in non-dopaminergic systems in response to the diminished availability of dopamine [71]. With [^18^F]CPFPX, a phase- and region-specific pattern of A_1_R expression in Huntington’s disease was detected, providing evidence that adenosinergic targets are involved in the pathophysiology of this disease [72]. More recently, the first partial agonist PET tracer, [^11^C]MMPD (Fig. 2, Table 2), was evaluated in rat brain [54]. It showed suitable blood–brain barrier (BBB) permeability, high specificity, and subtype selectivity in vivo. This finding may open new routes to visualize receptor occupancy of agonists or partial agonists at the A_1_R in drug development.

**Table 2:** Recent AR radioligands used for clinical PET or SPECT imaging

Radioligands	*K*_*D*_ (nM)^a^				Functionality	Ref
	A_1_	A_2A_	A_2B_	A_3_		
A_1_						
[^18^F]CPFPX	1.3	940	N.D.	N.D.	Antagonist	[51]
[^11^C]MPDX	4.2 (r)	> 100 (r)	N.D.	N.D.	Antagonist	[51]
[^11^C]MMPD	0.5	71	75	42% (1 μM)	Partial agonist	[54]
A_2A_						
[^11^C]TMSX or [^11^C]KF18446	1600 (r)	5.9 (r)	N.D.	N.D.	Antagonist	[55]
[^11^C]SCH442416	1.1	0.05	> 10,000	> 10,000	Antagonist	[56]
[^11^C]preladenant	> 1000	1.1	> 1700	> 1000	Antagonist	[57]
[^18^F]FESCH	43% (10 μM)	12	N.D.	60% (10 μM)	Antagonist	[58]
[^18^F]FPSCH	1000	54	N.D.	1320	Antagonist	[59]
[^18^F]MNI-444	N.D.	2.8	N.D.	N.D.	Antagonist	[60]
[^123^I]MNI-420	N.D.	2.0	N.D.	N.D.	Antagonist	[60]
A_2B_						
[^11^C]”4”	230	548	210	N.A.	Antagonist	[61]
[^18^F]”7a”	19	55	4.2	796	Antagonist	[62]
A_3_						
[^18^F]FE@SUPPY	4030	1720	N.D.	6.0	Antagonist	[63]
[^76^Br]MRS3581	N.D.	N.D.	N.D.	0.63	Agonist	[64]
[^76^Br]MRS5147	N.D.	N.D.	N.D.	0.62	Antagonist	[64]

*N.D.* not determined, *N.A.* not active

^a^The data are *K*_*D*_ values of radiolabeled compounds for human adenosine receptors unless otherwise indicated (r = rat) or % inhibition at the indicated concentration in brackets

### PET/SPECT tracers for the adenosine A_2A_ receptor

Several radioligands for PET imaging of cerebral A_2A_Rs have been introduced since the 1990s. The initial design of PET tracers for the A_2A_R started from xanthine-based antagonists, leading to the discovery of [^11^C]TMSX (Fig. 2, Table 2), previously abbreviated as [^11^C]KF18446. Though in vivo imaging of the human brain in healthy controls and in patients with Parkinson’s disease (PD) was relatively successful [73, 74], these xanthine derivatives are prone to photoisomerization, and thus [^11^C]TMSX could only be applied in PET scans under dimmed light. To circumvent this limitation, the first non-xanthine-based PET tracer, [^11^C]SCH442416 (Fig. 2, Table 2), was designed based on a known precursor, SCH58261. An increased binding potential of [^11^C]SCH442416 was observed in the striatum of Parkinson’s patients with levodopa-induced dyskinesias (LIDs), providing evidence that A_2A_R is a potential pharmacological target for the management of LIDs [75]. Since the problem of high non-specific binding (and consequential low target-to-non-target ratios) still remains for these ligands [76], Zhou et al. incorporated the ^11^C-radionuclide into clinical candidate preladenant. PET imaging in rats showed a high uptake of [^11^C]preladenant (Fig. 2, Table 2) in the striatum and low uptake in other regions of the brain, consistent with cerebral A_2A_ distribution [77]. Using [^11^C]preladenant in clinical PET studies, receptor occupancy by istradefylline, an approved A_2A_R antagonist, was measured in patients with Parkinson’s disease. It was demonstrated that istradefylline binds to A_2A_R in a dose-dependent manner, consequently resulting in near-maximal (94%) occupancy in the ventral striatum, thus establishing the dosage regimen of such CNS drugs [78]. Subsequently, to benefit from the prolonged half-life of these tracers, ^18^F-labeled A_2A_R antagonist PET tracers have been investigated for human studies. For example, two fluorine-18 labeled SCH442416 analogs, [^18^F]FESCH (Fig. 2, Table 2) and [^18^F]FPSCH (Fig. 2, Table 2), were reported as PET tracers used to image the A_2A_R in rat brain [79]. [^18^F]FESCH and [^18^F]FPSCH showed identical striatum-to-cerebellum ratios (4.6 at 37 min and 25 min post-injection, respectively), similar to the ratio obtained with [^11^C]SCH442416. Other examples are preladenant-based ligands, including a SPECT tracer, [^123^I]MNI-420 (Fig. 2, Table 2), and a PET ligand, [^18^F]MNI-444 (Fig. 2, Table 2). Both have been successfully applied in A_2A_R imaging studies in the human brain [80, 81]. [^123^I]MNI-420 rapidly entered the human brain and showed the highest specific binding in the striatum, consistent with known A_2A_R densities. [^18^F]MNI-444 showed an improved binding potential in the brain compared to [^11^C]TMSX and [^11^C]SCH442416, opening up the possibility to more broadly use in vivo A_2A_ PET imaging in neuroscience research.

### PET tracers for the adenosine A_2B_ receptor

So far only two radioligands for use in in vivo studies have been developed for A_2B_R, namely 1-[^11^C]”4” (Fig. 2, Table 2) and -[^18^F]”7a” (Fig. 2, Table 2) [61, 62]. The first compound, featuring a triazinobenzimidazole scaffold with moderate potency (IC_50_ = 210.2 ± 12.3 nM) towards A_2B_R, has been applied in PET studies in rats and showed the highest uptake in brown adipose tissue, lungs, and testes [61]. With a high chemical stability and good pharmacokinetic profile, this tool compound represented a good lead for the development of A_2B_R radiotracers. The second A_2B_R PET tracer was developed on a pyrazine-based antagonist with the potential to penetrate the blood–brain barrier [62]. Despite poor selectivity (A_2A_/A_2B_ = 13, A_1_/A_2B_ = 5), this radiolabeled ligand was further evaluated for its in vivo pharmacokinetic profile, revealing the formation of a radio-metabolite capable of penetrating the blood–brain barrier. With these PET studies, the stage is set for further A_2B_R probe design to enhance their selectivity and metabolic stability.

### PET tracers for the adenosine A_3_ receptor

The first PET tracer for A_3_R was developed by radiofluorination of FE@SUPPY (Fig. 2, Table 2), a selective and potent antagonist for hA_3_R [82, 83]. Although it had already been shown for the parent compound that the affinity for rat A_3_R was 140-fold lower than for human A_3_R, [^18^F]FE@SUPPY was studied for its biodistribution in rats, and specific binding in the rat brain was demonstrated using autoradiography [83]. A further preclinical PET study using [^18^F]FE@SUPPY to image A_3_R revealed a pronounced uptake in xenografted mice injected with cells overexpressing human A_3_R. This “humanized animal model” inspired to evaluate [^18^F]FE@SUPPY in mice xenografted with a human colorectal cancer cell line (HT-29) overexpressing A_3_R as a tumor marker. Unfortunately, this study to visualize the A_3_R in vivo was unsuccessful, presumably due to insufficient uptake of [^18^F]FE@SUPPY in the tumors, poor conservation of target expression in xenografts, or unfavorable pharmacokinetics of the tracer in mice [63]. In analogy to this, [^18^F]FE@SUPPY:2 (Fig. 2, Table 2) was developed by transforming the fluoroethylester into a fluoroethylthioester [84]. While a higher specific radioactivity was obtained ([^18^F]FE@SUPPY:2 = 340 ± 140 GBq/mol and [^18^F]FE@SUPPY = 70 ± 26 GBq/mol), the uptake pattern for the two PET tracers is distinct. Especially, brain to blood ratios are remarkably increased over time for [^18^F]FE@SUPPY, whereas those for [^18^F]FE@SUPPY:2 stayed unaltered. Lastly, a pair of structurally similar ligands (i.e., agonist MRS3581 and antagonist MRS5147) were reported as [^76^Br]-labeled potential PET radiotracers [64]. Both ligands showed similar biodistribution in rats, i.e., primarily uptake in the organs of metabolism and excretion. However, the uptake of agonist [^76^Br]MRS3581 (Fig. 2, Table 2) was an order of magnitude faster than that of antagonist [^76^Br]MRS5147 (Fig. 2, Table 2), possibly due to the presence of a uronamide group in the agonist to influence its bioavailability and permeation in vivo. In contrast, the antagonist [^76^Br]MRS5147 demonstrated an increased uptake in rat testes, an A_3_R-rich tissue, suggesting that the antagonist may also serve as a viable diagnostic molecular probe for pathological conditions with increased A_3_R expression.

## Fluorescent probes

As an alternative to radiolabeled molecular probes, fluorescent ligands have also been included into the pharmacological toolbox. This approach avoids the safety concerns associated with the disposal of radioisotopes and also provides the opportunity of a “real-time” readout of the ligand–receptor interaction. Fluorescent ligands for GPCRs are usually designed by incorporating an organic fluorophore, such as a BODIPY, AlexaFluor®, rhodamine, or NBD (nitrobenzoxadiazole) moiety into an existing GPCR agonist or antagonist pharmacophore via a linker. The use of these fluorescent probes in GPCR research has recently been reviewed [85] and includes studies on receptor localization, function, and regulation, but also on ligand–target binding kinetics, thus contributing to a detailed understanding of receptor physiology and pathophysiology. In addition, the development of newer methods and techniques, such as scanning confocal microscopy, fluorescence polarization, fluorescence correlation spectroscopy, resonance energy transfer (FRET or BRET), and flow cytometry, is boosting the potential use of fluorescent probes in drug discovery. The development of fluorescent ligands to characterize adenosine receptors has been the subject of intense investigation, which has been summarized in detail by Kozma et al. in 2013 [86]. Here, we will therefore summarize and review emerging fluorescent ligands for more recent applications on ARs.

### Fluorescent ligands for the adenosine A_1_ receptor

**Fig. 3:**
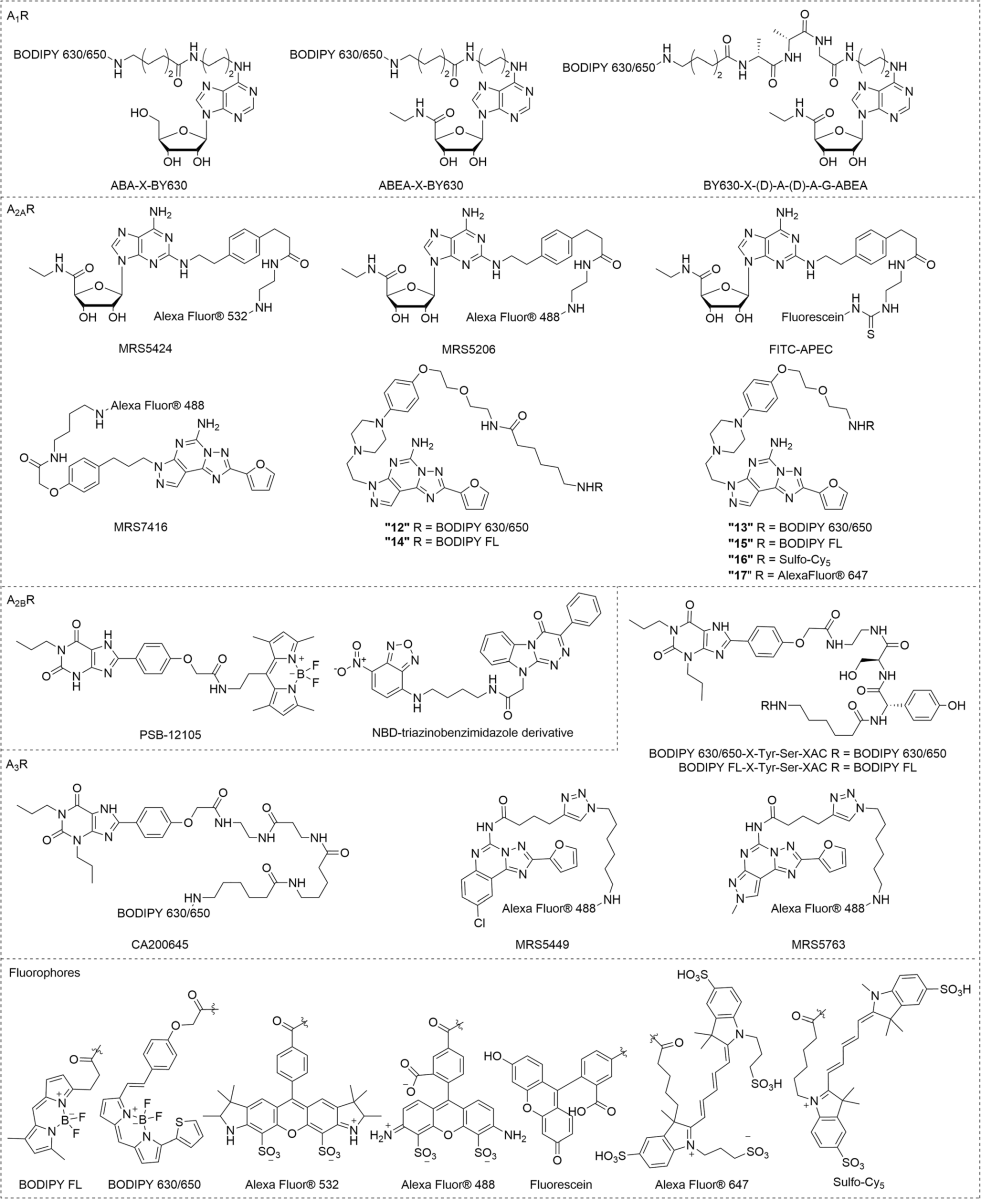
Chemical structures of recent fluorescent tools for ARs

To monitor ligand binding to receptors on the surface of living cells, a nano-luciferase (NanoLuc) BRET methodology (NanoBRET) has recently been established [87–89]. This approach was also applied to a study of allosteric modulators in intact living cells using fluorescent A_1_R agonists, such as the adenosine-based agonist, ABA-X-BY630 (Fig. 3, Table 3), and two NECA-based ligands, ABEA-X-BY630 (Fig. 3, Table 3) and BY630-X-(D)-A-(D)-A-G-ABEA (Fig. 3, Table 3) [90]. The two positive allosteric modulators tested were shown to increase the specific binding of the fluorescent A_1_R agonists, indicative for a switch of the A_1_R population to a more active receptor conformation.

**Table 3:** Recent AR fluorescent ligands

Ligands		*K*_*i*_/*K*_*D*_^a^				Functionality	Ref
		A_1_	A_2A_	A_2B_	A_3_		
A_1_	CA200645	34	N.D.	N.D.	6.2	Antagonist	[87, 89]
	ABA-X-BY630	589	N.D.	N.D.	N.D.	Agonist	[90]
	ABEA-X-BY630	1023	N.D.	N.D.	N.D.	Agonist	[90]
	BY630-X-AAG-ABEA	676	N.D.	N.D.	N.D.	Agonist	[90]
A_2A_	FITC-APEC	N.D.	57 (bovine)	N.D.	N.D.	Agonist	[91, 92]
	MRS7416	1680	30	N.D.	32% (10 μM)	Antagonist	[93]
	“12”	0^b^	41	0^b^	0^b^	Antagonist	[94]
	“13”	0^b^	41	0^b^	0^b^	Antagonist	[94]
	“14”	0^b^	17	0^b^	0^b^	Antagonist	[94]
	“15”	0^b^	22	0^b^	0^b^	Antagonist	[94]
	“16”	0^b^	83	0^b^	0^b^	Antagonist	[94]
	“17”	0^b^	60	0^b^	0^b^	Antagonist	[94]
A_2B_	PSB-12105	≥ 10,000	> 10,000	1.8	> 10,000	Antagonist	[95]
	NBD-derivative	1380	> 10,000	20%^c^ (10 μM)	> 10,000	Antagonist	[96]
A_3_	BODIPY 630/650-X-Tyr-Ser-XAC	24	N.D.	N.D.	0.76	Antagonist	[97, 98]
	BODIPY FL-X-Tyr-Ser-XAC	316	N.D.	N.D.	11	Antagonist	[97, 98]
	MRS5449	87	73	N.D.	6.4	Antagonist	[99]
	MRS5763	N.D.	90	N.D.	32	Antagonist	[100]

*N.D.* not determined

^a^*K*_*i*_/*K*_*D*_ values for compounds for the indicated human adenosine receptors

^b^Specific BRET ratio on respective (NanoLuc-labeled) adenosine receptors

^c^% of cAMP production induced by 100 nM of NECA in CHO cells expressing human A_2B_R at 10-nM concentration compound

### Fluorescent ligands for the adenosine A_2A_ receptor

MRS5424 (Fig. 3, Table 3) is a fluorescent adduct of agonist APEC with AlexaFluor®532. Using this probe, allosteric modulation within A_2A_R/D_2_R heterodimers was followed using real-time FRET [101]. A negative allosteric effect on A_2A_R ligand binding and receptor activation was found when the D_2_R agonist quinpirole was added. This heterodimer interaction was further validated in a higher-throughput flow cytometry–based assay with the fluorescent agonist MRS5206 (APEC-AlexaFluor® 488) (Fig. 3, Table 3) [102]. These experiments provided evidence for a differential D_2_R-mediated negative allosteric modulation of A_2A_R agonist binding, in particular for apomorphine, a drug used in the treatment of PD. Recently, using a fluorescence polarization assay, McNeely et al. employed a fluorescent agonist, FITC-APEC (Fig. 3, Table 3), to characterize the binding kinetics of three hA_2A_R ligands [91, 92]. The kinetic parameters of these unlabeled ligands, computed using a numerical solution approach, showed good consistency with those determined in a conventional radioligand binding assay.

Endeavors to enhance selectivity towards hA_2A_R and improve the physicochemical properties of fluorescent ligands led to the discovery of MRS7416 (Fig. 3, Table 3), which is based on the antagonist SCH442416 [93]. As a fluorescent tracer, MRS7416 displayed low non-specific binding at hA_2A_R in flow cytometry experiments. From molecular docking studies, the researchers suggested that the fluorescent AlexaFluor® 488 moiety present in MRS7416 is binding to the hydrophilic extracellular loops of the receptor. This would make the probe essentially “bitopic,” i.e., bridging two separate domains of the hA_2A_R. Very recently, the toolbox was expanded with a series of preladenant-based ligands equipped with a range of fluorophores [94]. These compounds showed p*K*_*D*_ values between 7.1 and 7.8 and were highly A_2A_-selective with practically no binding to the other adenosine receptor subtypes.

### Fluorescent ligands for the adenosine A_2B_ receptor

The first selective A_2B_ fluorescent ligand reported, PSB-12105 (Fig. 3, Table 3), was synthesized by integrating a BODIPY moiety into the pharmacophore of 8-substituted xanthine derivatives [95]. Besides fluorescently labeling CHO cells expressing recombinant human A_2B_R, this ligand was used to establish an A_2B_R binding assay on living cells in a flow cytometry setup. Barresi et al. reported on another series of (non-selective) fluorescent antagonists for labeling A_1_Rs and A_2B_Rs [96]. In one of the ligands, a fluorescent group, NBD (Fig. 3, Table 3), was linked to a triazinobenzimidazole scaffold. This fluorescent antagonist showed a clear labeling of bone marrow–derived mesenchymal stem cell membranes, which was largely prevented by pre-incubation with selective agonists for A_1_R and A_2B_R. These findings provide a sound basis for the design of novel fluorescent ligands to monitor the expression and localization of A_2B_R in living cells.

### Fluorescent ligands for the adenosine A_3_ receptor

The non-selective A_1_R/A_3_R antagonist, CA200645, was employed as a tool compound to develop a robust competition binding assay to, e.g., screen for new chemical templates and fragments for A_3_R at a live cell high-content screening system [87, 88]. Besides, CA200645 was also applied to study the A_3_R localization on intact human neutrophils. It appeared that A_3_R activation induces the formation of filipodia-like extensions and bacterial phagocytosis [103]. Modification of the linker component in CA200645 by the insertion of a dipeptide yielded two A_3_-selective fluorescent ligands, BODIPY 630/650-X-Tyr-Ser-XAC (Fig. 3, Table 3) and BODIPY FL-X-Tyr-Ser-XAC (Fig. 3, Table 3) [97]. Both ligands showed displaceable membrane binding with little non-specific binding in a fluorescent confocal microscopy setup. Additionally, these ligands were applied in a NanoBRET-based assay to study the kinetic aspects of ligand binding [98]. A similar strategy to incorporate a (three amino acid) peptide linker was applied to an existing non-selective adenosine-based fluorescent agonist, ABEA-X-BY630, yielding the highly potent fluorescent agonist BY630-X-(*D*)-Ala-(*D*)-Ala-Gly-ABEA at A_3_R [104]. This probe was used to visualize the internalization of YFP-tagged as well as -untagged receptors, and appeared to promote the formation of intracellular receptor–arrestin-3 complexes. In addition, click chemistry serves as a versatile approach to simplify compound synthesis, as it provides the means for facile incorporation of fluorescent tags. CGS15943, a triazolo-quinazoline antagonist scaffold, was extended with an alkyne moiety to be click-conjugated with AlexaFluor® 488, yielding a selective A_3_R fluorescent probe, MRS5449 (Fig. 3, Table 3) [99]. In flow cytometry, this molecular probe was used to quantify hA_3_R and to perform ligand screening in intact cells. The most recent addition to the A_3_R toolbox has been a series of pyrazolo[4,3-e]-1,2,4-triazolo[1,5-c]pyrimidine derivatives equipped with fluorescein-based fluorophores FITC and AlexaFluor® 488 [100]. The best compound from this series (MRS5763) exhibits a reasonable affinity of 32 nM on the *h*A_3_R and has some selectivity towards the *h*A_2A_R.

## Covalent ligands

Another class of molecular probes is formed by covalent ligands. The term covalent here refers to the ability of these compounds to bind the receptor irreversibly by forming a covalent bond to a specific amino acid residue located at or near the ligand binding site [105]. Depending on the type of covalent interaction induced, some different considerations are made concerning the design of these compounds. Generally, high affinity and selectivity for the target receptor will increase receptor occupancy and decrease non-specific or off-target binding, thus improving specific covalent labeling [106]. Two types of covalent ligands have been developed until now: electrophilic and photo-reactive ligands. Choosing the correct functional group (or warhead) that can react with the amino acid residues present in the binding site is essential for successful covalent probe design. Photo-reactive ligands possess a light-sensitive group, such as aryl azide, diazirine, or benzophenone, which is irradiated with light of a specific wavelength to yield highly reactive nitrene, carbene, or benzophenone-derived diradicals. These reactive species subsequently form a covalent bond with a neighboring amino acid residue through a variety of insertion reactions [107]. Photo-reactive ligands, occasionally combined with mass spectrometry, have been applied in GPCR research to determine the binding site of ligands and to identify the partner receptor for orphan ligands [108]. When combined with a radioactive label, photoaffinity probes emerge, which are used to study GPCR localization using autoradiography [109]. Electrophilic ligands on the other hand possess a reactive electrophile as a warhead, such as (iso)thiocyanate, sulfonyl fluoride, or a Michael acceptor like acrylamide. These electrophiles react with nucleophilic amino acid residues such as lysine, serine, and cysteine near the binding site of the ligand. When combined with in silico modeling and site-directed mutagenesis studies, these chemo-reactive ligands often enable characterization of the GPCR ligand binding site. Additionally, electrophilic covalent ligands have been applied to study receptor reserve, turnover, and subtype discrimination [110, 111]. Lastly, binding of a covalent ligand stabilizes the receptor into an active or inactive conformation, which in turn facilitates crystallization of the receptor–ligand complex. This aids in structural biology studies using X-ray diffraction or cryoEM, providing valuable insights into the structure and function of GPCRs [112]. A prime example of this is the case of the human adenosine A_1_ receptor, which was recently co-crystallized with covalent antagonist DU172 [3]. There are numerous reported covalent ligands for adenosine receptors that have in some way contributed to the characterization of these receptors and their ligand binding sites. These ligands will be summarized below, and their applications will be discussed.

### Covalent ligands for the adenosine A_1_ receptor

**Fig. 4:**
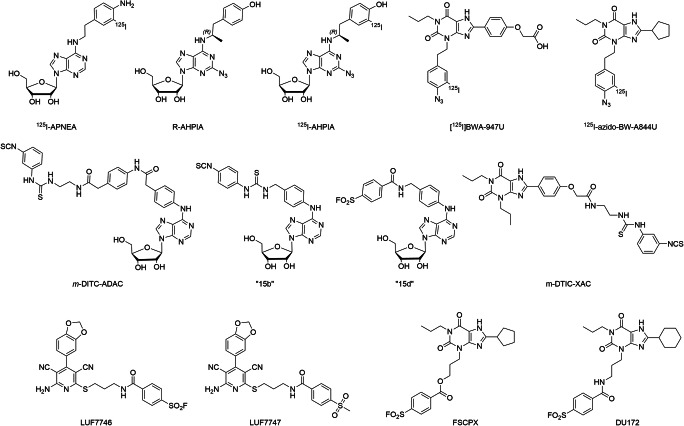
Chemical structures of covalent ligands for A_1_R. LUF7747 is a reversible control ligand for LUF7746

Arguably, the first example of photoaffinity labeling of an adenosine receptor dates back to 1985 when N^6^-2-(4-aminophenyl)ethyladenosine (APNEA), a non-selective adenosine-based agonist with high affinity for both A_1_R and A_3_R, was coupled to the A_1_R [113]. In an attempt to characterize the A_1_R structure, radioiodinated ^125^I-APNEA (Fig. 4, Table 4) was incubated with A_1_R and reacted with crosslinking reagent *N*-hydroxysuccinimidyl 6-(4-azido-2-nitrophenylamino)hexanoate (SANPAH) in situ. Subsequent UV irradiation resulted in a 38-kDa protein being covalently labeled with the radioligand in rat cerebral cortex and adipocyte membranes. Since this process was completely blocked by co-incubating with a selective A_1_R agonist, this protein was designated as A_1_R. Strictly speaking, this radioactive ligand is obviously not inherently photo-reactive and thus not a photoaffinity probe per se. Interestingly, in the same year, efforts to develop an inherently photo-reactive ligand based on the *R*-PIA scaffold, one of the most selective A_1_R agonists, were successful. A photoactivatable azido group was positioned at the purine core structure, generating the photolabile ligand *R*-AHPIA (Fig. 4, Table 4) [129]. It exhibited similar affinity (*K*_*i*_ = 1.5 nM) and efficacy (EC_50_ = 35 nM) as its parent compound, *R*-PIA, but after photoactivation, it showed irreversible inhibition of approximately 40% of the receptor binding sites. Such covalent labeling of A_1_R led to a concentration-dependent reduction of cellular cAMP levels, consistent with activation of rA_1_R and correlating with receptor occupancy [130]. Similar to the case of APNEA, when *R*-AHPIA was radioiodinated to yield ^125^I-AHPIA (Fig. 4, Table 4), SDS-PAGE analysis of rat brain membranes that were incubated with this covalent radioligand and UV-irradiated showed the appearance of a single protein band of ~ 35 kDa [129]. Interestingly, even though *R*-AHPIA is about 60-fold selective for the A_1_R, it is also a partial agonist at the A_2A_R, and pretreatment with *R*-AHPIA reduced the stimulatory effect of NECA, indicating persistent binding of the ligand and subsequent reduced activation by a full agonist [131]. In the search for covalent antagonists, 4-azidophenethyl xanthine derivative [^125^I]BW-A947U (Fig. 4) was synthesized, and optimization (analogous to the development of selective A_1_R antagonist DPCPX) yielded the next photoactivatable antagonist, ^125^I-azido-BW-A844U (Fig. 4, Table 4) [114, 132, 133]. Both ligands are xanthine-based antagonists that have a light-sensitive aryl azide located on the xanthine 3-position. Photoaffinity labeling of partially purified receptor with ^125^I-azido-BW-A844U followed by chemical or enzymatic fragmentation experiments demonstrated that the covalently modified amino acids were located at transmembrane domain III of the A_1_R. This approach provided clear insight into the amino acids surrounding the binding pocket of the A_1_R and thus aided in the development of three-dimensional models of the receptor.

**Table 4:** Covalent ligands for adenosine receptors

Ligands		Apparent IC_50_/*K*_*i*_/*K*_*D*_ (nM)^a^				Functionality	Ref
		A_1_	A_2A_	A_2B_	A_3_		
A_1_	^125^I-APNEA	2.0 (r)	N.D.	N.D.	N.D.	Agonist	[113]
	*R*-AHPIA	1.6 (r)	N.D.	N.D.	N.D.	Agonist	[113]
	^125^I-AHPIA	2.0 (r)	N.D.	N.D.	N.D.	Agonist	[113]
	^125^I-azido-BW-A844U	0.14 (b)	N.D.	N.D.	N.D.	Antagonist	[114]
	*p*-DITC-ADAC	0.47 (r)	191 (r)	N.D.	N.D.	Agonist	[115]
	*m*-DITC-ADAC	0.87 (r)	176 (r)	N.D.	N.D.	Agonist	[115]
	LUF7746	4.0	26% (1 μM)	26% (1 μM)	25% (1 μM)	Partial agonist	[116]
	*m*-DITC-XAC	2.4 (r)	343 (r)	N.D.	N.D.	Antagonist	[115]
	FSCPX	12	1200	N.D.	N.D.	Antagonist	[117]
	DU172	21	2.8	N.D.	N.D.	Antagonist	[117]
A_2A_	^125^I-azido-PAPA-APEC	N.D.	1.2	N.D.	N.D.	Agonist	[118]
	[^125^I]AzPE	N.D.	1.7	N.D.	N.D.	Agonist	[119]
	“9”	N.D.	40	N.D.	N.D.	Agonist	[120]
	*p*-DITC-APEC	276 (r)	35 (r)	N.D.	N.D.	Agonist	[121]
	MRS5854	500	23	N.D.	207	Agonist	[122]
	MRS5854-azide	30% (10 μM)	4360	N.D.	1810	Agonist	[122]
	ISC	20,300	111	N.D.		Antagonist	[123]
	LUF7445	372	1.0	0% (1 μM)	49	Antagonist	[124]
	LUF7487	19	1.5	N.D.	60	Antagonist	[6]
A_3_	MRS1163	145 (r)	272 (r)	N.D.	10.0 (r)	Agonist	[125]
	SO_2_F-MRS1191	41% (100 μM, r)	20% (100 μM, r)	N.D.	2.4	Antagonist	[126]
	SO_2_F-MRE-3008-F20	< 5% (100 nM)	50	N.D.	79% (100 nM)	Antagonist	[127]
	LUF7602	794	1300	0% (10 μM)	10	Antagonist	[128]

*N.D.* not determined

^a^The data are apparent affinities (nM) for the human adenosine receptors or % displacement at the concentration in brackets unless indicated otherwise (r = rat, b = bovine)

Initial attempts in the development of chemo-reactive agonist ligands for the A_1_R were focused on functionalizing the adenosine scaffold with isothiocyanates or sulfonyl fluorides to serve as warheads [115, 134]. In the first reported case, *p*- and *m*-DITC-ADAC (Fig. 4, Table 4), both adenosine derivatives with nanomolar affinity substituted on the N^6^-position with an isothiocyanate-bearing linker, were synthesized and tested on the A_1_R [135]. At nanomolar concentration, both ligands irreversibly occupied approximately half of the A_1_R binding sites. In a functional cAMP accumulation assay, both agonists elicited a sustained, antagonist-insensitive, A_1_R-mediated response. Since the incorporation of a warhead via the N^6^-position of the adenosine scaffold was well tolerated and showed no negative effect on the ligands’ affinities, a series of adenosine derivatives bearing diverse linker types and warheads were synthesized and examined. Two promising compounds, isothiocyanate 15b and sulfonyl fluoride 15d (Fig. 4, Table 4), were validated as irreversible agonists promoting persistent A_1_R-mediated guanine nucleotide exchange activity in a manner resistant to both agonist and antagonist addition [134]. Furthermore, these two ligands demonstrated their capacity to thermo-stabilize purified, detergent-solubilized A_1_R in a ThermoFluor assay to a significantly higher degree than the high-affinity agonist NECA could. These thermostabilized receptors with covalently bound ligands allowed purification of the receptor in a monodisperse state, which greatly facilitated structure determination by X-ray crystallography [134]. Very recently, our group reported a capadenoson derivative, which was equipped with a fluorosulfonyl warhead to give LUF7746, a non-ribose (dicyanopyridine-based) partial agonist for the A_1_R [116]. This compound was shown to selectively bind the A_1_R in a time-dependent manner with an apparent affinity (at 4 h pre-incubation) in the low-nanomolar range. Additionally, LUF7746 was compared to LUF7747, a non-reactive methylsulfonyl control compound, which showed no time-dependent binding. Interestingly, whereas both compounds showed an intrinsic activity with *E*_max_ around 60%, which was also demonstrated in a label-free whole cell assay, only the effect of LUF7747 could be diminished by the addition of antagonist DPCPX. The ability of LUF7746 to persistently activate the receptor was largely abolished by performing site-directed mutagenesis (Y271F^7.36^) to remove the tyrosine’s reactive hydroxyl, indicating the importance of this conserved amino acid in the covalent interaction. With respect to chemo-reactive antagonists, two approaches have been explored, both starting from the xanthine scaffold. The first class comprises the 8-substituted 1, 3-dipropylxanthines [136]. One such compound is *m*-DITC-XAC (Fig. 4, Table 4), an isothiocyanate derivative of the relatively non-selective AR antagonist XAC. It was found to be a potent A_1_R antagonist in rat brain (*K*_*i*_ = 2.4 nM) and was used to study the receptor reserve in guinea pig atrioventricular nodes [137]. In the second approach, the electrophilic fluorosulfonyl group was placed on the 3-position of the xanthine core, as was done in covalent tool FSCPX (Fig. 4, Table 4) [138]. This compound had a good affinity for the A_1_R (IC_50_ = 10 nM), and treatment with 10- or 50-nM FSCPX led to reductions in the available A_1_R binding sites of 60% and 74%, respectively. In a follow-up study, it was demonstrated that FSCPX irreversibly antagonized cardiac A_1_R-mediated responses. Subsequently, it was shown that FSCPX was unable to significantly decrease the maximal direct inotropic response to four A_1_R full agonists (NECA, CPA, CHA, and adenosine) in guinea pig atria, which demonstrated a considerable A_1_R reserve for direct negative inotropy [139]. In in vivo experiments, FSCPX was used successfully as a “receptor knock-down” tool when IV infusion of FSCPX in conscious rats attenuated CPA-mediated bradycardia [140]. As the ester bond present near the warhead of FSCPX is prone to hydrolysis, a follow-up structural modification was performed with a focus on linker types [117, 141]. This resulted in a closely related analog with improved stability, DU172 (Fig. 4, Table 4). The affinity of DU172 (IC_50_ = 25 nM) was in line with that of FSCPX, and pretreatment of DDT_1_ MF2 cells with DU172 resulted in a concentration-dependent decrease in the A_1_R binding sites, indicating that it behaved as an irreversible ligand indeed. This covalent ligand–receptor interaction has been the basis for the structure elucidation of A_1_R due to improved receptor stability [3].

### Covalent ligands for the adenosine A_2A_ receptor

**Fig. 5:**
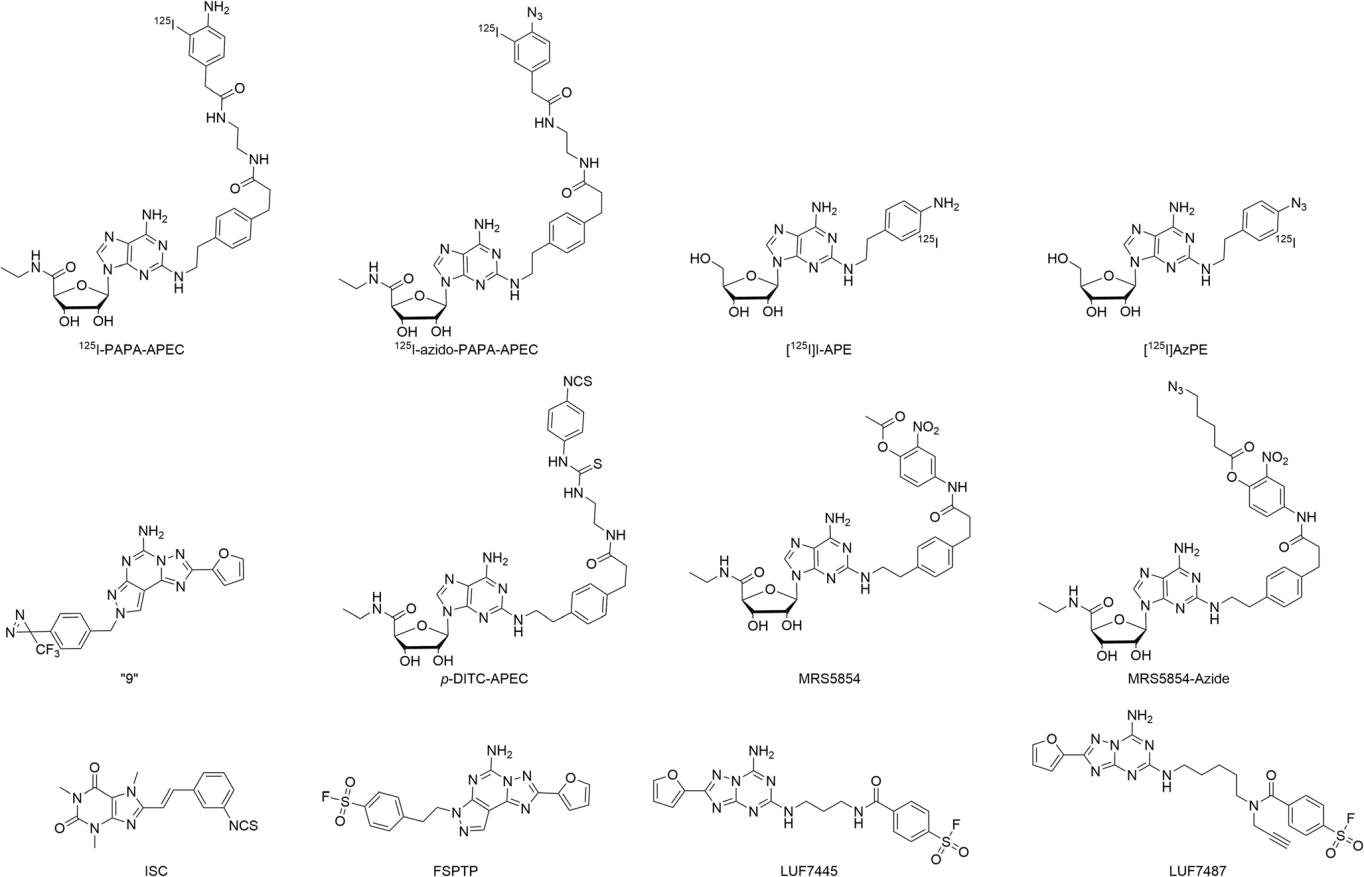
Chemical structures of covalent ligands for A_2A_R

For the A_2A_R, initial characterization of the receptor was aided by a radioiodinated analog of APEC, a prototypical ribose-based selective A_2A_R agonist. Similar to the initial A_1_R studies, ^125^I-PAPA-APEC (Fig. 5, Table 4) was cross-linked to the A_2A_R in bovine striatal membranes using SANPAH and was shown to covalently label a 45-kDa protein [121, 142]. Both NECA and R-PIA were able to prevent the covalent labeling of the 45-kDa protein by ^125^I-PAPA-APEC, providing evidence that this protein is the A_2A_R indeed. Subsequently, the photoactivatable azido analog ^125^I-azido-PAPA-APEC (Fig. 5, Table 4) was developed and was used to directly label the same 45-kDa protein in bovine striatal membranes with 3-fold greater efficiency of photo-incorporation [118]. A further characterization of the binding domain was performed by Piersen et al., who performed photoaffinity labeling of the canine A_2A_R overexpressed in COS M6 cells with ^125^I-azido-PAPA-APEC and tracked the cross-linked transmembrane domain V [143]. However, no individual amino acid residues responsible for the covalent interaction were identified. These studies were later repeated with a novel adenosine-based radioligand [^125^I]I-APE, which showed less hydrophobic interactions than ^125^I-PAPA-APEC and had higher specific radioactivity than [^3^H]CGS21680 [119]. Its azido analog, [^125^I]AzPE (Fig. 5, Table 4), showed saturable, high-affinity binding in rabbit striatal membranes (*K*_*D*_ = 1.7 nM), and photolabeling identified a protein of 45 kDa that displayed the appropriate pharmacology of the A_2A_R. More recently, photoaffinity labeling has been combined with mass spectrometry analysis to map detailed ligand–receptor binding sites. Muranaka et al. started from the not-so-A_2A_R-selective SCH58261 scaffold [144] and incorporated the trifluoromethyl diazirine group to yield photoaffinity ligand 9 (Fig. 5, Table 4) [120]. When purified hA_2A_R was photolabeled with this ligand and subjected to protease digestion, cross-link positions were identified with LC-MS/MS. The most likely amino acid candidate for this ligand was Y271^7.36^ in transmembrane domain VII. This is the first reported case in which the cross-linked amino acid was elucidated by mass spectrometry, which demonstrates the power of combining mass spectrometry–based proteomics and covalent labeling in the elucidation and characterization of GPCR ligand binding sites.

Analogous to the photo-reactive ligands, APEC also served as a parent ligand for the initial design of chemo-reactive ligands for A_2A_R. One exemplary compound is *p*-DITC-APEC (Fig. 5, Table 4), which has a reactive 4-isothiocyanatophenyl residue attached to the C-2 substituent of the purine ring [121]. It had good affinity (*K*_*i*_ = 7.1 nM at bovine A_2A_R) [121] and, at a concentration of 100 nM, irreversibly blocked 77% of [^3^H]CGS21680 binding in rabbit striatal membranes [145]. In isolated, perfused guinea pig hearts, treatment with *p*-DITC-APEC caused a prolonged, persistent, and concentration-dependent coronary vasodilatation, which is evidence of an irreversible activation of A_2A_R [146]. More recently, an APEC analog bearing an active 2-nitrophenyl ester was synthesized (MRS5854, Fig. 5, Table 4). This ligand was designed to bind to the receptor irreversibly and subsequently transfer its terminal acyl group to a nucleophilic amino acid residue on extracellular loop 2 (ECL2) of the A_2A_R [122]. This acyl transfer would prevent the ECL2-lysine-mediated recognition of ligands, effectively blocking the receptor. Pre-incubation of hA_2A_R with MRS5854 followed by extensive washing indeed showed near-complete inhibition of radioligand binding. When ECL2-lysine K153 was mutated to an alanine residue, a partial restoration of *B*_max_ was observed after treatment with MRS5854, confirming that K153 is the anchor point for the covalent interaction. Interestingly, the *K*_*D*_ for the radioligand used ([^3^H]ZM241385) was not significantly influenced by this mutation, indicating that the targeted lysine residue is not important for ligand binding and that acyl transfer seems to prevent binding by blocking entry to the binding pocket instead of preventing the recognition of ligands. In parallel, the active acyl was replaced by an azido-pentanoate group to generate MRS5854-azide. Although this ligand showed diminished affinity towards the A_2A_R, it nevertheless caused a slight reduction in *B*_max_, suggesting that at least part of the receptors was covalently labeled with the azido-pentanoate. This azido group would theoretically allow for click-ligation to functionalized alkynes; however, applications have not yet been reported.

Three approaches have been taken to develop electrophilic covalent probes for the A_2A_R. The first example is ISC (Fig. 5, Table 4), an isothiocyanate-functionalized xanthine-based antagonist for A_2A_R, which irreversibly binds to 80% of rA_2A_R at 20 μM [123]. A second approach yielded FSPTP (Fig. 5, Table 4), the *para*-fluorosulfonyl derivative of SCH58261, which was used to investigate the level of A_2A_R reserve [147]. More recently, our research group used the molecular structure of the antagonist ZM241385 as a starting point for the design of a third electrophilic covalent ligand. This endeavor yielded LUF7445 (Fig. 5, Table 4), a potent fluorosulfonyl-equipped antagonist with an apparent affinity for the hA_2A_R in the nanomolar range (p*K*_*i*_ = 8.99) [124]. Aided by site-directed mutagenesis studies, it was shown that LUF7445 binds to K153^ECL2^, the same residue that was also involved in the acyl transfer of covalent agonist MRS5854. After optimization of the chemical structure, the most potent ligand was retained for further structural modification and was equipped with an alkyne click handle (adjacent to the warhead), resulting in the bifunctional probe LUF7487 (Fig. 5, Table 4) [6]. This affinity-based probe made it possible to visualize the receptor on SDS-PAGE via click-ligation with a sulfonated Cy-3 fluorophore. The hA_2A_R was successfully labeled in cell membranes, making LUF7487 a promising tool compound that sets the stage for the further development of probes to study GPCRs. The development of affinity-based probes may open the door for the identification and target validation of GPCRs in a more native environment.

### Covalent ligands for the adenosine A_3_ receptor

**Fig. 6:**
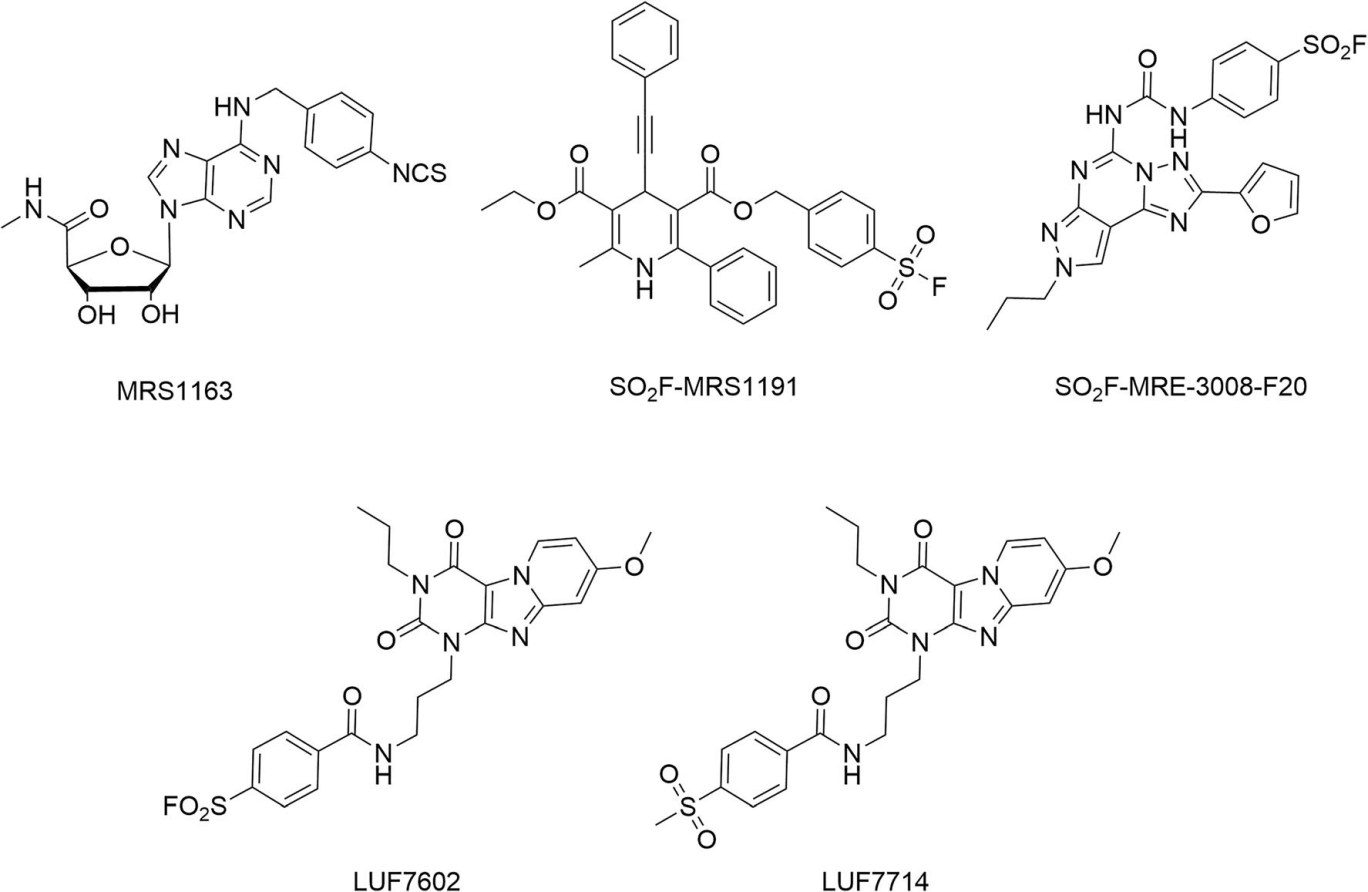
Chemical structures of covalent ligands for A_3_R. LUF7714 is a reversible control ligand for LUF7602

While there are no photo-reactive or chemo-reactive ligands available for the A_2B_R, the case for the A_3_R is also still rather minimal. No photo-reactive ligands and only four “classes” of chemo-reactive ligands are available for the A_3_R. MRS1163 (Fig. 6, Table 4), the only irreversibly binding agonist for the A_3_R, was derived from the selective A_3_R agonist IB-MECA [125]. It features a chemo-reactive isothiocyanate moiety, which replaced the iodine substituent on IB-MECA, and showed an apparent *K*_*i*_ value in the low-nanomolar range (10 nM), which is comparable to IB-MECA. Treatment of rA_3_R with 100 nM of MRS1163 led to a 41% loss in the available receptor binding sites, and its irreversible nature was demonstrated by the lack of recovery of A_3_R binding sites after extensive washing. Using a “functionalized congener approach,” the Jacobson group developed an electrophilic antagonist for the A_3_R based on the 1,4-dihydropyridine template, a selective A_3_R scaffold. A fluorosulfonyl-substituted phenyl group was installed on MRS1191, thereby generating the functionalized congener SO_2_F-MRS1191 (Fig. 6, Table 4) [126]. It was reported to possess improved affinity (2.4 nM) over the corresponding sulfonamide compound (292 nM). When 100 nM of SO_2_F-MRS1191 was incubated with hA_3_R-transfected HEK-293 cell membranes, approximately 56% of the hA_3_R binding sites were irreversibly occupied. A second covalent antagonist was generated based on MRE-3008-F20, a highly potent and selective A_3_R antagonist [127]. By replacing the methoxy group in MRE-3008-F20 with a sulfonyl fluoride moiety, an irreversibly binding derivative, SO_2_F-MRE-3008-F20 (Fig. 6, Table 4), was synthesized. At a concentration of 100 nM, SO_2_F-MRE-3008-F20 inhibited binding of the radioligand [^125^I]I-AB-MECA by 79%. By docking the ligand in a homology model of the A_3_R, it was speculated that two amino acids, Cys251 or Ser247, are the most probable binding partners for covalent interaction. Recently, our group also designed covalent antagonists for the hA_3_R [128]. A series of tricyclic xanthine–derived ligands bearing a fluorosulfonyl warhead and varying linkers was synthesized. The most potent ligand, LUF7602 (Fig. 6, Table 4), had high affinity for the hA_3_R (*K*_*i*_ = 10 nM). Additionally, a non-reactive methylsulfonyl derivative LUF7714 was developed as a reversible control compound. A series of assays, comprising of time-dependent affinity determination, washout experiments, and [^35^S]GTPγS binding assays, then validated LUF7602 as a covalent antagonist. Based on homology docking, tyrosine Y265^7.36^ was identified as potential covalent anchor, and when this residue was mutated to phenylalanine, the mutant receptor displayed a significant decrease in affinity for LUF7602 (IC_50_ = 16 nM for hA_3_R-WT, IC_50_ = 1000 nM for hA_3_R-Y265^7.36^F), while the affinity of LUF7714 (IC_50_ = 1259 nM for hA_3_R-WT, IC_50_ = 1000 nM for hA_3_R-Y265^7.36^F) was unaltered. It is worth mentioning that this particular tyrosine residue is conserved among adenosine receptors and is also the anchor point of DU172 and LUF7746, the aforementioned covalent antagonist and partial agonist for the hA_1_R [117]. Hence, this tyrosine residue potentially represents a universal anchor point for covalent probes designed for adenosine receptors. In general, covalent probes, supported by molecular modeling and site-directed mutagenesis, can serve as powerful tools to characterize the spatial orientation and topography of ligand–receptor binding sites.

## Concluding remarks

Molecular probes, including radioligands and fluorescent and covalent ligands, are important tool compounds that facilitate the biochemical and structural investigation of GPCRs. As shown in this review, these probes provide information about the nature of adenosine receptors, next to a deeper understanding of receptor regulation and the pathological and physiological roles of this GPCR subfamily. In particular, when combined with other techniques such as receptor mutagenesis, X-ray crystallography, and homology modeling, these tools provide a powerful platform for molecular receptor pharmacology.

Radioligands are the most developed tools for GPCRs. An established standard radioligand binding assay provides crucial and reliable measurements of GPCRs interacting with their synthetic ligands as well as newly developed probes. Binding of an agonist radioligand may reveal different apparent affinity states depending on the receptor states (i.e., G protein–coupled and G protein–uncoupled) or cell-dependent effector coupling; agonist binding often labels the G protein–coupled (“active”) state of the receptor only. Thus, antagonist radioligands are generally considered more acceptable in receptor classification than agonists. Among the adenosine receptors, there is still an urgent need for the development of antagonist radioligands for the A_2B_R and A_3_R with high affinity (*K*_*D*_ values of 1 nM or less), low non-specific binding, and better selectivity. For in vivo assays, the development of PET ligands targeting A_2B_R and A_3_R has still been limited to receptor occupancy studies, biodistribution, or pharmacokinetic characterization, while PET ligands for A_1_R and A_2A_R have blossomed in clinical studies, particularly for neurological disorders. Studies on A_2B_R and A_3_R are generally considered to be hampered by the low expression level of these receptors in endogenous tissue, insufficient affinity of the tool compound, and unclear mechanisms involved in receptor function. It is anticipated that continued efforts to develop high-affinity and selective PET tracers for adenosine receptors will further our understanding of the role these receptors have in disease conditions.

Concerns about radiation safety and shelf life have fueled the continuing interest in small-molecule fluorescent tools. Recent examples summarized in this review demonstrate that fluorescent probes represent an alternative approach to investigate AR characteristics. However, their use is still sub-optimal due to the often high level of non-specific membrane binding brought by the hydrophobic pharmacophore and fluorophore. Hence, researchers should pay more attention to designing probes with favorable physicochemical properties. Besides, the in vivo applications of such tools are still hampered, partly due to their short excitation wavelengths and low tissue penetration [148]. Future development of synthetic ligands with a focus on near-infrared (NIR) fluorophores might be advantageous, especially since such wavelengths are not harmful to cells and have a relatively low absorption. NIR probes have already been employed to study the cannabinoid CB_2_ and α_1_-adrenergic receptors [149, 150]. Depending on the intended goal and applicability domain, careful consideration of the pros and cons of fluorescent or radiolabeled compounds (Table 5) is essential.

**Table 5:** Major pros and cons of radioligands versus fluorescent ligands

Radioligands		Fluorescent ligands	
Pro	Con	Pro	Con
Established standard assays	Radiation concerns	No radiation/safety issues, easy handling	Target often engineered
Highly sensitive	Limited shelf life	Shelf stable	Non-specific binding, sensitivity
In vivo use (PET ligands)	Limited “real-time” readout	“Real-time” measurements	Limited in vivo applicability
Commercial availability	Safety issues, waste handling	Application in microscopy setup	Tag size

Compared to radioligand and fluorescent probes, covalent ligands do not possess any detectable functionality for direct quantification or visualization of receptors. However, when combined with site-direct mutagenesis, mass spectrometry, and peptide sequencing, they constitute a powerful approach compared to classic reversible ligands to study adenosine receptor subtype and structure, map ligand binding sites, investigate the physiological and pathological roles of receptors, and determine the correlation between receptor occupancy and response (Table 6). The emergence of the activity-based protein profiling technique inspired researchers to equip probes with click handles to yield bifunctional probes that can be used to visualize receptors for target validation. In this strategy, a probe binds the receptor with less perturbation compared to relatively large tags linked to ligand scaffolds beforehand, which bridges the field of chemical biology with the field of molecular pharmacology to better investigate receptor–ligand interactions. In future research, different tags may be introduced; for instance, a biotin tag would allow for streptavidin-mediated receptor enrichment followed by LC/MS analysis. Of note, the A_2B_R has been known as the more poorly characterized adenosine receptor subtype. This also has limited the development of molecular probes targeting A_2B_R specifically, in particular for covalently binding ligands, where no case has been reported so far. Covalent probes for A_2B_R and A_3_R may also assist in the structure elucidation of these two adenosine receptor subtypes, which are currently still lacking.

**Table 6:** Major pros and cons of covalent versus reversible ligands

Covalent		Reversible	
Pro	Con	Pro	Con
Permanently bind to the receptor, increased target occupancy	Risk of non-specific binding to other, lower-affinity targets	Can be washed out of binding pocket	Binding to receptor is temporary
Enable “chemical biology” approaches	Inherently reactive, and thus unstable	No reactive group, more stable	Target occupancy influenced by binding kinetics
Stabilize receptor for, e.g., crystallization	Artificial interaction unlike endogenous ligands	Interaction more similar to endogenous ligands	Orthosteric ligands in competition with endogenous ligands, surmountable effect

For decades, scientists have been continuously developing tool compounds to study adenosine receptors. In this endeavor, the use of covalent or reversible probes, whether radiolabeled or fluorescent, has been instrumental (i) to discover new chemical entities, (ii) to characterize and interrogate adenosine receptor subtypes both in vitro and in vivo, and (iii) to study their behavior in physiological and disease conditions. This review has summarized evidence for these applications, but hopefully, it also serves as an invitation to walk another mile to further improve probe characteristics and develop additional tags that allow the investigation of adenosine receptors and other GPCRs in even finer detail.
